# Drug induced exfoliative dermatitis: state of the art

**DOI:** 10.1186/s12948-016-0045-0

**Published:** 2016-08-22

**Authors:** Mona-Rita Yacoub, Alvise Berti, Corrado Campochiaro, Enrico Tombetti, Giuseppe Alvise Ramirez, Andrea Nico, Elisabetta Di Leo, Paola Fantini, Maria Grazia Sabbadini, Eustachio Nettis, Giselda Colombo

**Affiliations:** 1 Department of Allergy and Clinical Immunology, IRCCS San Raffaele Hospital, Via Olgettina 60, 20132 Milan, Italy; 2 Vita-Salute San Raffaele University, Milan, Italy; 3 Section of Allergy and Clinical Immunology, Dept. of Internal Medicine, University of Bari, Bari, Italy

**Keywords:** Exfoliative dermatitis, Drug hypersensitivity, Stevens–Johnson syndrome, Lyell’s syndrome, Toxic epidermal necrolysis, Erythema multiforme, Delayed type hypersensitivity, Pathogenesis, Clinical features, Therapy

## Abstract

Drug induced exfoliative dermatitis (ED) are a group of rare and severe drug hypersensitivity reactions (DHR) involving skin and usually occurring from days to several weeks after drug exposure. Erythema multiforme (EM), Stevens–Johnson syndrome (SJS) and toxic epidermal necrolysis (TEN) are the main clinical presentations of drug induced ED. Overall, T cells are the central player of these immune-mediated drug reactions. Here we provide a systematic review on frequency, risk factors, pathogenesis, clinical features and management of patients with drug induced ED.

**Electronic supplementary material:**

The online version of this article (doi:10.1186/s12948-016-0045-0) contains supplementary material, which is available to authorized users.

## Background

Cutaneous drug eruptions are one of the most common types of adverse reaction to medications, with an overall incidence of 2–3 % in hospitalized patients [1]. In particular, drug induced exfoliative dermatitis (ED) are a group of rare and more severe drug hypersensitivity reactions (DHR) involving skin and mucous membranes and usually occurring from days to several weeks after drug exposure [2]. Erythema multiforme (EM), Stevens–Johnson syndrome (SJS) and toxic epidermal necrolysis (TEN) are the main clinical presentations of drug induced ED. Important data on ED have been obtained by RegiSCAR (European Registry of Severe Cutaneous Adverse Reactions to Drugs: www.regiscar.org), an ongoing pharmaco-epidemiologic study conducted in patients with SJS and TEN. Overall, T cells are the central player of these immune-mediated drug reactions. Immune-histopathological features allow to distinguish generalized bullous drug eruption from SJS/TEN [3–6]. Still, treatment indication, choice and dosage remain unclear, and efficacy yet unproven. Here we provide a systematic review of frequency, risk factors, molecular and cellular mechanisms of reactions, clinical features, diagnostic work-up and therapy approaches to drug induced ED.

## Epidemiology

Epidemiological studies on EM, SJS and TEN syndromes report different results, probably related to several biases, such as ethnical differences, diagnostic criteria and drug consumption patterns in different socio-economic systems. Albeit the lack of epidemiologic data regarding EM, its reported prevalence is less than 1 % [7–10]. Several authors report the incidence of hospitalization for EM ranging from 0.4–6 cases per million people per year of northern Europe [11] to almost 40 cases per million people per year of United States [12]. EM usually occurs in young adults of 20–40 years of age [13], with women affected more frequently than men (1.5:1.0) [14]. Recurrence occurs in around one-third of cases [15] and there is a genetic predisposition for certain Asian groups [16]. Mucosal involvement could achieve almost 65 % of patients [17]. EM’s mortality rate is not well reported.

Overall, incidence of SJS/TEN ranges from 2 to 7 cases per million person per year [9, 18–20], with SJS the commonest [21]. In HIV patients, the risk of SJS and TEN have been reported to be thousand-fold higher, roughly 1 per 1000 per year [19]. Prevalence is low, with mortality of roughly 5–12.5 % for SJS and 50 % for TEN [1, 2]. In general, they occur more frequently in women, with a male to female ratio of 0.6 [22]. The overall mortality rate is roughly 30 %, ranging from 10 % for SJS to more than 30 % for TEN, with the survival rate worsening until 1 year after disease onset [9, 18–21].

Pharmacogenetics studies have found an association between susceptibility to recurrent EM in response to several stimuli and human leukocyte antigen (HLA) haplotypes of class II, in particular HLA DQB1*0301 [23].

On the other hand, it has been demonstrated that genetic predisposition may increase the risk for sulphonamide-induced [24] and carbamazepine-induced TEN and SJS [25]. Scientific evidences suggest a role for HLAs and drug-induced SJS/TEN, although some racial differences have been found that can be due to variation of frequencies of these alleles and to the presence of other susceptibility genes [26]. These studies have confirmed an association between carbamazepine-induced SJS/TEN with HLA-B*1502 allele among Han Chinese [27], carbamazepine and HLA-A*3101 and HLA-B*1511 [16], phenytoin and HLA-B*1502 [28], allopurinol and HLA-B*5801 [29]. For carbamazpine, several studies have found a common link between specific HLAs and different kinds of cutaneous adverse reactions, as for HLA-A*3101 in Japanese [30] and Europeans [31]. Because a certain degree of cross-reactivity between the various aromatic anti-epileptic drugs exists, some HLAs have been found to be related to SJS/TEN with two drugs, as the case of HLA-B*1502 with both phenytoin and oxcarbazepine [32].

## Pathogenesis

Apoptosis-inducing factors and lymphocyte-mediated cytotoxicity have been deeply investigated in ED. Four main pathways have been found to play important roles in the pathogenesis of keratinocyte death: (1) Fas-FasL interaction, (2) Perforin/granzyme B pathway, (3) Granulysin and (4) Tumor necrosis factor *α* (TNF-*α*) [26].

1. *Fas*-*FasL interaction*: Fas is a membrane-bound protein that after interaction with Fas-ligand (FasL) induces a programmed cell death, through the activation of intracellular caspases. T and NK lymphocytes can produce FasL that eventually binds to target cells. In ED increased levels of FasL have been detected in patients’ sera [33]. The exact source of FasL production has not been yet identified as different groups have postulated that the production might be sought in keratinocytes themselves [33] or in peripheral blood mononuclear cells [34]. In any case all authors concluded that the blockage of FasL prevents keratinocyte apoptosis [35]. The exact role of FasL in the pathogenesis of toxic epidermal necrolysis is still questionable especially because a correlation between serum FasL levels and disease severity has not been established and because its levels have been found to be increased also in drug-induced hypersensitivity syndrome and maculopapular eruption [36].
2. *Perforin/granzyme* B pathway: Nassif and colleagues have proposed a role for perforin/grazyme B in keratinocyte death [37]. They found that the inhibition of these molecules could attenuate the cytotoxic effect of lymphocytes toward keratinocytes. A correlation between increased levels of perforin/granzyme B and the severity of TEN was also described [38].
3. *Granulysin*: Granulysin is a pro-apoptotic protein that binds to the cell membrane by means of charge interaction without the need of a specific receptor, producing a cell membrane disruption, and leading to possible cell death. Chung and colleagues found an high expression of this molecule in TEN blister fluid [39] and confirmed both in vitro and in vivo its dose-dependent cytotoxicity [39]. Moreover, after granulysin depletion, they observed an increase in cell viability. The serum levels of granulysin were also found to be increased in the early stage of SJS/TEN, but not in other cutaneous DHR [40].
4. *Tumor necrosis factor α*: TNF-α seems also to play an important role in TEN [41]. The fluid of blisters from TEN patients was found to be rich in TNF-α, produced by monocytes/macrophages present in the epidermis [42], especially the subpopulation expressing CD16, known to produce higher levels of inflammatory cytokines [43]. TNF-*α* has a dual role: interacts with TNF-R1 activating Fas pathway and activates NF-κB leading to cell survival. Although the final result of this dual interaction is still under investigation, it seems that the combination of TNF-α, IFN-γ (also present in TEN patients) and the activation of other death receptors such as TWEAK can lead to apoptosis of keratinocytes [44].

A central role in the pathogenesis of ED is played by CD8+ lymphocytes and NK cells. Even though there is not a significant increase in the number of T cells infiltrating the skin of TEN patients, it was found that their role is crucial, even more than HLAs types. In fact, it was demonstrated that the specificity of the TCR is a required condition for the self-reaction to occur. In particular, a specific T cell clonotype was present in the majority of patients with carbamazepine-induced SJS/TEN and that this clonotype was absent in all patients tolerant to the drug who shared the same HLA with the SJS/TEN patients [45]. The enhanced activation of CD8 T cells seems also to be influenced by the impaired function of CD4 + CD25 + FoxP3 + Treg cells found in the peripheral blood of TEN patients in the acute phase [46].

In addition to all these mechanisms, alarmins, endogenous molecules released after cell damage, were found to be transiently increased in SJS/TEN patients, perhaps amplifying the immune response, including α-defensin, S100A and HMGB1 [47]. These molecules may play a role in amplifying the immune response and in increasing the release of other toxic metabolites from inflammatory cells [48].

## Histologic features

Given the different histopathological features of the EM, SJS and TEN, we decided to discuss them separately.

### EM

In EM a lymphocytic infiltrate (CD8+ and macrophages), associated with vacuolar changes and dyskeratosis of basal keratinocytes, is found along the dermo-epidermal junction, while there is a moderate lymphocytic infiltrate around the superficial vascular plexus [20]. Partial to full thickness epidermal necrosis, intraepidermal vesiculation or subepidermal blisters, due to spongiosis and to the cellular damage of the basal layer of the epidermis, can be present in the advanced disease [49] Occasionally, severe papillary edema is also present [20]. The dermis shows an inflammatory infiltrate characterized by a high-density lichenoid infiltrate rich in T cells (CD4+ more than CD8+) with macrophages, few neutrophils and occasional eosinophils; the latter especially seen in cases of DHR [5, 50].

### SJS

The SJS histology is characterized by a poor dermal inflammatory cell infiltrate and full thickness necrosis of epidermis [20, 49]. The epidermal-dermal junction shows changes, ranging from vacuolar alteration to subepidermal blisters [20]. The dermo-epidermal junction and epidermis are infiltrated mostly by CD8+ T lymphocytes whereas dermal infiltrate, mainly made from CD4+ T lymphocytes, is superficial and mostly perivascular [20, 51].

### TEN

TEN is characterized by full-thickness epidermal necrosis with an evident epidermal detachment and sloughing caused by necrosis of keratinocytes following apoptosis [49, 52]. It’s also characterized by a cell-poor infiltrate, where macrophages and dendrocytes with a strong TNF-α immunoreactivity predominate [6, 50].

## Clinical manifestations and culprit agents

**Fig. 1:**
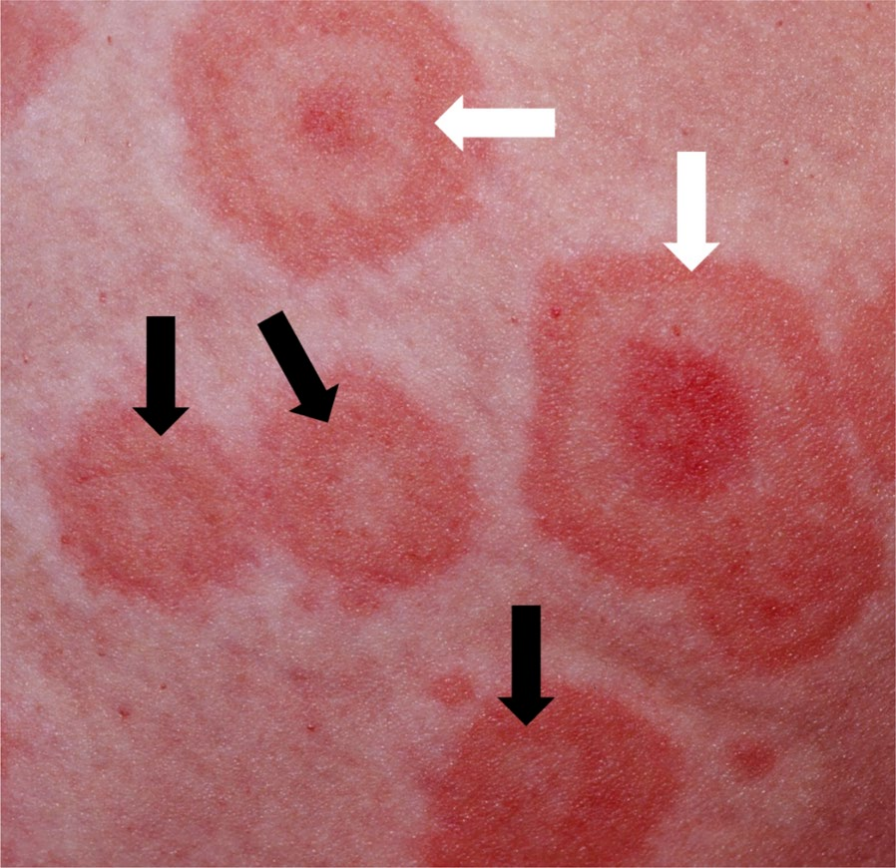
Erythema multiforme (photo reproduced with permission of Gary White, MD): typical target lesions (*white arrows*) together with atypical two-zoned lesions (*black arrows*)

EM is a self-limited skin condition mainly associated with infections and drugs [53, 54]. It has a wide spectrum of severity, and it is divided in minor and major (EMM). The former is usually a recurring, localized eruption of the skin characterized by pathognomonic target or iris lesions, with minimal or no mucosal involvement (Fig. 1). EMM is a clinically severe, potentially life-threatening, extensive sloughing of epidermis, generally involving mucosal tissue. In EMM lesions typically begin on the extremities and sometimes spread to the trunk. Infectious agents are the major cause of EM, in around 90 % of cases, especially for EM minor and in children. Herpes simplex virus (HSV) 1 and 2 are the main triggers in young adults (>80 % of cases), followed by Epstein-Barr virus (EBV), and Mycoplasma pneumonia [55–58]. Among drug related cases, the main triggering factors are sulfonamides, nonsteroidal anti-inflammatories (NSAIDs), penicillins, and anticonvulsants (Table 1) [59]. Neoplastic conditions (renal and gastric carcinoma), autoimmune disease (inflammatory bowel disease), HIV infection, radiation, and food additives/chemicals have been reported to be predisposing factor [59].

**Table 1:** Most common culprit drugs in SJS/TEN and EM

Drugs associated with Stevens–Johnson syndrome and toxic epidermal necrolisis				
	Risk a priori	Prevalence in SJS/TEN registries	Extension of employment	Probable/very probable causality in multicenter trials
Allopurinol	Very high	Very high	Widespread	Frequent
Anticonvulsants Carbamazepine Lamotrigine Phenobarbital Phenytoin Valproic Acid	Very high	Very high	Widespread	Frequent
NSAIDs	Variable	High	Widespread	Variable
Oxicam NSAIDs	Very high	Low	Limited	Frequent
Sulfonamides Cotrimoxazole Sulfadiazine Sulfasalazine Others	High	High	Widespread	Frequent
*Non-sulfa antibiotics*				
Aminopenicillins	Low	Medium	Widespread	Non frequent
Cephalosporins	Medium	Medium	Widespread	Non frequent
Quinolones	Medium	Medium	Widespread	Moderately frequent
Macrolides	Medium	Medium	Widespread	No
Tetraciclines	Medium	Low	Medium	Frequent
Nevirapine	High	High	Limited	Frequent
Pantoprazole	Unknown	Low	Widespread	ND
Paracetamol	Low	High	Widespread	Non freqeuent
Furosemide	Low	Variable	Widespread	ND
Sertraline	High	Low	Medium	Frequent
*Drugs associated with erythema multiforme*				
Sulfonamides				
NSAIDs				
Anticonvulsants				
Antibiotics (mainly penicillins)				

SJS and TEN are two overlapping syndromes resembling severe burn lesions and characterized by skin detachment. When less than 10 % of the body surface area (BSA) is involved, it is defined SJS, when between 10 and 30 % of BSA it is defined overlapping SJS/TEN, when more than 30 % of BSA, TEN [2] (Additional file 1: Figure S1, Additional file 2: Figure S2). SJS/TEN syndrome is associated with severe blistering, mucocutaneous peeling, and multi-organ damage and could be life threatening. TEN is also known as “Lyell syndrome”, since it was first described by Alan Lyell in 1956 [2, 60].

Unlike EMM, SJS and TEN are mainly related to medication use. The strength of association with the development of SJS/TEN may vary among countries and historical periods, reflecting differences in ethnicities and prescription habits among the studied populations [61–64]. Exposure to anticonvulsivants (phenytoin, phenobarbital, lamotrigine), non-nucleoside reverse transcriptase inhibitors (nevirapine), cotrimoxazole and other sulfa drugs (sulfasalazine), allopurinol and oxicam NSAIDs [2] confers a higher risk of developing SJS/TEN. Several authors reported also an increased incidence for aminopenicillins, cephalosporins, and quinolones [61, 62]. Drugs such as paracetamol, other non-oxicam NSAIDs and furosemide, bringing a relatively low risk of SJS/TEN a priori, are also highly prevalent as putative culprit agents in large SJS/TEN registries, due to their widespread use in the general population [63, 64] (Table 1). Rarely, *Mycoplasma pneumoniae*, dengue virus, cytomegalovirus, and contrast media may be the causative agent of SJS and TEN [22, 65–67].

It is not completely clear whether EM and SJS are separate clinical entities or if they represent two different expressions of a single disease process. However, according to a consensus definition [54], EMM syndrome has been separated from SJS/TEN spectrum.

## Diagnosis E prognosis

### Prodromal and acute phase

During the acute reaction, diagnosis of ED is mainly based on clinical parameters. Initial symptoms could be aspecific, as fever, stinging eyes and discomfort upon swallowing, occurring few days before the onset of mucocutaneous involvement. Early sites of skin involvement include trunk, face, palms and soles and rapidly spread to cover a variable extension of the body. EMM is characterizes by target lesions, circular lesions of 1-2 cm of diameter, that are defined as typical or atypical that tends to blister. Typical target lesions consist of three components: a dusky central area or blister, a dark red inflammatory zone surrounded by a pale ring of edema, and an erythematous halo on the periphery. Atypical target lesions manifest as raised, edematous, palpable lesions with only two zones of color change and/or an extensive exanthema with a poorly defined border darker in the center (Fig. 1). In SJS and TEN mucosal erosions on the lips, oral cavity, upper airways, conjunctiva, genital tract or ocular level are frequent [60, 68–70].

### Allergy workout

In acute phase it is crucial to assess the culprit agent, in particular when the patient was assuming several drugs at time of DHR. First of all, Sassolas and coauthors proposed an algorithm of drug causality (ALDEN) in order to improve the individual assessment of drug causality in TEN and SJS [71]. ALDEN has shown a good accuracy to assess drug causality compared to data obtained by pharmacovigilance method and case–control results of the EuroSCAR case–control analysis for drugs associated with TEN.

#### In vivo tests

Diagnosis in a routine setting is based on patch test (PT) while skin test (prick and intradermal tests) with a delayed reading are contraindicated in these patients [72]. PTs have to be performed at least 6 months after the recovery of the reaction, and show a variable sensitivity considering the implied drug, being higher for beta-lactam, glycopeptide antibiotics, carbamazepine, lamotrigine, proton pump inhibitors, tetrazepam, trimethoprim—sulfametoxazole, pseudoephedrine and ramipril [73–76].

#### In vitro tests

Lymphocyte transformation test (LTT) performed as described by Pichler and Tilch [77] shows a lower sensitivity in severe DHR compared to less severe DHR [78] but, if available, should be performed within 1 week after the onset of skin rash in SJS and TEN [79]. A promising and complementary in vitro tool has been used by Polak ME et al. [80], which consists of the determination of IFNγ and IL4 by ELISpot (Enzyme-linked immunospot assay), allowing to increase the sensitivity of LTT during acute DHR (82 versus 50 % if compared to LPA).

### Prognosis

**Fig. 2:**
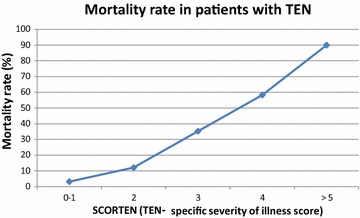
Mortality rate of patients with TEN has shown to be directly correlated to SCORTEN. Adapted from Ref. [81]

A severity-of-Illness score for toxic epidermal necrolysis (SCORTEN) has been proposed and validated to predict the risk of death at admission [81]. The SCORTEN scale is based on a minimal set of parameters as described in the following table. For the calculation, available values on vital and laboratory parameters within the first 3 days after admission to the first hospital are considered when the reaction started outside the hospital (community patients) or at the date of hospitalization for in-hospital patients. Considered variables in SCORTEN are shown in Table 2. Mortality rate of patients with TEN has shown to be directly correlated to SCORTEN, as shown in Fig. 2.

**Table 2:** The SCORTEN variables

SCORTEN variables	
Age ≥40 years	1
Involved BSA at day 1 ≥10 %	1
Presence of cancer or malignancy	1
Heart rate ≥120 beats per minute	1
Serum urea level ≥10 mmol/L	1
Serum bicarbonate level <20 mmol/L	1
Serum glucose ≥14 mmol/L	1

### Differential diagnosis

As described in Table 3, major differential diagnosis of EM and SJS/TEN are (1) staphylococcal scalded skin syndrome (SSSS), (2) autoimmune blistering diseases and disseminated fixed bullous drug eruption, (3) others severe delayed DHR [6, 70, 82] (4) Graft versus host disease

- *SSSS* is characterized by periorificial face scabs, de-epithelialization of friction zones and conspicuous desquamation after initial erythroderma. Trigger is an exotoxin released by *Staphylococcus aureus* [83]. A useful sign for differential diagnosis is the absence of mucosal involvement, except for conjunctiva. Main discriminating factors between EMM, SJS, SJS-TEN, TEN and SSSS is summarized in Table 3 [84].
- *Pemphigus vulgaris, paraneoplastic pemphigus, bullous pemphigoid and linear IgA dermatosis* have to be considered. In order to rule out autoimmune blistering diseases, direct immune fluorescence staining should be additionally performed to exclude the presence of immunoglobulin and/or complement deposition in the epidermis and/or the epidermal-dermal zone, absent in ED. The Nikolsky’s sign is not specific for SJS/TEN, in fact it is present also in auto-immune blistering diseases like pemphigus vulgaris. *Bullous pemphigoid* is characterized by large, tense bullae, but may begin as an urticarial eruption. Linear IgA dermatosis most commonly presents in patients older than 30 years. The lesions consist of pruritic, annular papules, vesicles, and bullae that are found in groups, clinically it is similar to dermatitis herpetiformis, without a gluten-sensitive enteropathy [85]. Bullous dermatoses can be debilitating and possibly fatal. Pemphigus vulgaris usually starts in the oral mucosa followed by blistering of the skin, which is often painful. Paraneoplastic pemphigus is associated with neoplasms, most commonly of lymphoid tissue, but also Waldenström’s macroglobulinemia, sarcomas, thymomas and Castleman’s disease.
- *Other delayed DHR*
- Acute generalized exanthematous pustulosis (AGEP) is characterized by acute erythematous skin lesions, generally arising in the face and intertriginous areas, subsequently sterile pinhead-sized nonfollicular pustules arise and if they coalesce, may sometimes mimic a positive Nikolsky’s sign and in this case the condition may be misinterpreted as TEN [86].
- Drug reaction with Eosinophilia and systemic symptoms (DRESS) syndrome can mimic SJS and TEN in the early phases, since ED can occur together with the typical maculo-papular rash. In contrast with DRESS, eosinophilia and atypical lymphocytes are not described in patients with SJS or TEN. Both DRESS and SJS may have increased liver enzymes and hepatitis, but they occur in only 10 % of cases of SJS compared to 80 % of DRESS. Interstitial nephritis is common in DRESS syndrome, occurring roughly in 40 % of cases, whereas pre-renal azotemia may occur in SJS and TEN.
- *Graft versus host disease (GVHD)* Acute GVHD usually happens within the first 6 months after a transplant. Common acute symptoms include abdominal pain or cramps, nausea, vomiting, and diarrhea, jaundice, skin rash and eyes dryness and therefore could mimic the prodromal and early phase of ED. The diagnosis of GVDH requires histological confirmation [87].

**Table 3:** Differential diagnosis in a patient with suspected exfoliative dermatitis

Pathological condition	Pattern of skin lesions	Body surface area with epidermal detachment (%)	Trigger	Distribution of lesions
Erythema multiforme major (EMM)	Typical and atypical target papules and plaques, minimum involvement of mucous membranes (especially oral mucosae)	<10	Infection (*Mycoplasma pneumoniae*, *Herpes simplex*), drugs	Predominantly acrally distributed, i.e., begin on hands and feet
Stevens–Johnson syndrome (SJS)	No target lesions typical/atypical target lesions flattened, cotton wool spots purple confluent in the skin of the face and trunk, serious eruptions mucous membranes at the level of one or more sites	<10	Drugs	Diffuse. The eruption begins on the trunk
Overlap syndrome between Stevens–Johnson and Toxic Epidermal Necrolysis (SJS/TEN)	No target lesions/typical target lesions/atypical target lesions flattened	Between 10 and 30	Drugs	Diffuse. The eruption begins on the trunk
Toxic epidermal necrolysis (TEN)	No target lesions/typical target lesions/atypical target lesions flattened; begins with severe mucosal erosions and progresses to a detachment spread and generalized epidermis.	>30	Drugs	Diffuse. The eruption begins on the trunk
Staphylococcical scalded skin syndrome (SSSS)	Variable detachment between the stratum granulosum and the stratum corneum	Variable	Bacterial infection (*Staphylococci)*	Diffuse. No mucosal involvement except for conjunctiva

## Management and therapy

The therapeutic approach of EMM, SJS, TEN depends on extension of skin, mucosal involvement and systemic patient’s conditions. A multidisciplinary team is fundamental in the therapeutic management of patients affected by exfoliative DHR. The team should include not only physicians but also dedicated nurses, physiotherapists and psychologists and should be instituted during the first 24 h after patient admission. Patients present an acute high-grade of skin and mucosal insufficiency that obviously leads to great impairment in the defenses against bacteria that normally live on the skin, increasing the high risk of systemic infections. Moreover, transpiration and thermoregulation are greatly impaired with an elevated loss of fluids, proteins and electrolytes through the damaged skin and mucosae. For these reasons, patients should be admitted to intensive burn care units or in semi-intensive care units where they may have access to sterile rooms and to dedicated medical personnel [49, 88].

Patients can be extremely suffering because of the pain induced by skin and mucosal detachment. They usually have fever, are dyspneic and cannot physiologically feed. The most important actions to do are listed in Fig. 2, and described below.

### a) Immediate individuation and interruption of the culprit agent

As written before, Sassolas B. et al. [71] realized an algorhitm named ALDEN (algorithm of drug causality for epidermal necrolysis) which helps to establish a cause/effect relationship as “probable” or “very probable” in 70 % of cases. All non-indispensable drugs have to be stopped because they could alter the metabolism of the culprit agent.

### b) Evaluation of the skin and mucosal involvement

Dermatologist and/or allergist should confirm the diagnosis, individuate the culprit agent, give indications about skin management and necessity to obtain the consultation of the ENT specialist, the gynecologist/urologist, the ophthalmologist and/or the pulmonologist in the case of mucosal involvement.

### c) First-line interventions

Patient must be placed in an antidecubitus fluidized bed and room temperature must be kept at 30–32 °C in order to slow catabolism and reduce the loss of calories through the skin [89]. All the linen must be sterile. It is necessary to obtain as soon as possible a central venous access and to start a continuous monitoring of vital signs. In case of an oral mucositis that impairs nutrition, it is indicated to position a nasogastric tube. Also a vesical catheter should be placed to avoid urethral synechiae and to have a precise fluid balance. In case of a respiratory failure, oxygen should be administrated and a NIMV may be required. Temporary tracheostomy may be necessary in case of extended mucosal damage. In serious cases invasive ventilation can be necessary for ARDS. It is also extremely important to obtain within the first 24 h cultural samples from skin together with blood, urine, nasal, pharyngeal and bronchus cultures. Ophthalmologic consultations must be repeated at fixed intervals to avoid the appearance of conjunctival irreversible complications such as chronic conjunctivitis with squamous metaplasia, trichiasis, symblepharon, punctate keratitis and sicca syndrome. Gynecologist consultation is required for avoiding the appearance of vaginal phimosis or sinechias.

### d) Prophylactic, supportive and complications therapy

1. *Hydration and hemodynamic balance*. Fluid balance is a main focus. Once established the percentage of the involved skin, lactate Ringer infusion of 1–2 mL/Kg/ % of involved skin must be started during the first 24 h [91]. The velocity of infusion should be regulated according to patients arterial pressure with the aim of 30 mL/h urinary output (1 mL/kg/h in case of a child). Blood gas analysis, glucose and creatinine levels together with electrolytes should be evaluated and therapy should be modified accordingly. Vasoactive amines may be necessary in case of shock. Albumin is recommended only is albumin serum level is <2.5 mg/dL. Furosemide or ethacrynic acid may be required to maintain an adequate urinary output [90].
2. *Nutritional support*. An increased metabolism is typical of patients with extended disepithelizated areas. This hypermetabolic state is also furtherly increased by the inflammation present in affected areas. Early enteral nutrition has also a protective effect on the intestinal mucosa and decreases bacterial colonization. Usually the amount of calories is 1500–2000 kcal/day and the velocity of infusion is gradually increased based on patients tolerability [92].
3. *Gastric protection*. To avoid the appearance of gastric stress ulcer it is recommended to start a therapy with intravenous proton pump inhibitors.
4. *Anticoagulation therapy*. For the prevention of deep venous thrombosis; usually low molecular weight heparin at prophylactic dose are used.
5. *Antipyretic therapy.* It is recommended to use 1.5 mg/kg hydrocortisone. If necessary, it can be repeated every 6–8 h. NSAIDs should be avoided as they can induce ED as well.
6. *Painkiller therapy*. Intravenous administration is recommended. In more severe cases continuous iv therapy can be necessary. Most common used drugs are: morphine, fentanyl, propofol and midazolam.
7. *Antibiotic therapy*. It is not recommended to use prophylactic antibiotic therapy. It should be used only in case of a documented positivity of cultural samples. If there is a high suspicion of infection without a documented source of infection, broad range empiric therapy should be started.
8. *Antiviral therapy*. Ganciclovir and cidofovir should be used when polymerase-chain reactions (PCR) on peripheral blood or other biological sample identifies a viral reactivation (HHV6, HHV7, EBV and CMV). In more severe cases antiviral therapies should be given together with intravenous immunoglobulins [93].
9. *Growth-factors (G-CSF).* It recommended to used G-CSF in patients with febrile neutropenia [94, 95].
10. *Plasmapheresis*. It should be considered only once the patient is stable and if the skin damage is still ongoing and doesn’t respond to other conventional therapies (corticosteroids or IVIG). Plasmapheresis may have a role in the treatment of ED because it removes Fas-L [96], other cytokines known to be implied in the pathogenesis (IL-6, IL-8, TNF-α) [97, 98]. Moreover Mawson A and colleagues hypothesized that the efficacy of plasmapheresis is able to reduce serum level of vitamin A. In patients with SJS/TEN increased serum levels of retinoid acid have been found. These levels could reflect the interaction between culprit drugs and aldehyde dehydrogenase that is the enzyme which metabolizes retinoid acid. Increased level of retinoid acid could be responsible for keratinocytes apoptosis [99].
11. *Topical treatment*. Patients must be cleaned in the affected areas until epithelization starts. In spared areas it is necessary to avoid skin detachment. It is also recommended to void larger vesicles with a syringe. It is important to protect the damaged skin with sterile fat dressing especially in the genital area. 5 % silver nitrate compresses have antiseptic properties. Synthetic bilaminar membranes with silver nitrate have also a role in skin repairing and avoid protein loss through the damaged skin [100, 101]. It is advised against the use of silver sulfadiazine because sulphonamide can be culprit agents. Autologous transplantation of mesenchymal umbilical cord cells seems also to be highly efficacious [102]. Accurate eye cleaning with saline solution is fundamental for the prevention of synechiae and for reducing corneal damage. In more severe cases corneal protective lens can be used. It could also be useful to use artificial tears and lubricating antiseptic gels. The applications of topical cyclosporine and autologous serum have also been showed to be useful in refractory cases [103]. Oral hygiene with antiseptic and painkiller mouthwash (chlorhexidine + lidocaine + aluminum hydroxide) together with aerosol therapy with saline and bronchodilators can reduce upper airways symptoms.

### e) Anti-inflammatory and systemic immunosuppressive therapy

It is important to take into consideration the mechanism of action of the different drugs in the pathogenesis of ED [104].

Systemic corticosteroids: These are the most common used drugs because of their known anti-inflammatory and immunosuppressive effect through the inhibition of activated cytotoxic T-cells and the production of cytokines. Corticosteroids could also reduce the amount of keratinocytes apoptosis and the activation of caspases [105]. In EMM their efficacy is demonstrated in controlling the evolution of the disease [106]. In SJS, SJS/TEN and TEN the efficacy of corticosteroids is far from being demonstrated. Recently, a meta-analysis based on 6 retrospective studies evaluating the role of corticosteroids alone or together with IVIG has been published [107]. In this study, 965 patients were reviewed. The authors concluded that they couldn’t demonstrate corticosteroids efficacy in monotherapy, but the use of steroid alone is not linked to an increased risk of mortality due to infective complications [108, 109].

The most commonly used steroids were methylprednisolone, prednisolone and dexamethasone. The induction dosage in EMM is usually 1 mg/kg/day that should be maintained until a complete control of the skin is obtained. The taper of steroid therapy should be gradual [93]. In most severe cases the suggested dosage is iv 1–1.5 mg/kg/day. Iv bolus of steroid (dexamethasone 100–300 mg/day or methylprednisolone 250–1000 mg/day) for 3 consecutive days with a gradual taper steroid therapy is sometimes advised. A switch to oral therapy can be performed once the mucosal conditions improve.

If after 4 days there is not an improvement it is advised to consider the association of steroid or its replacement with one of the following drugs [49, 93]:

*Intravenous immunoglobulins (IVIG)*: play their role through the inhibition of Fas–Fas ligand interaction that it is supposed to be the first step in keratinocytes apoptosis [33]. A recently published meta-analysis by Huang [110] and coworkers on IVIG in SJS/SJS-TEN/TEN reviewed 17 studies with 221 patients and compared the results obtained with high-dosage IVIG (>2 g/kg) compared to lower-dosage IVIG (<2 g/kg). 12 out of 17 studies concluded for a positive role of IVIG in ED. In the 5 studies that concluded negatively for IVIG, the dosage was below 0.4 g/kg/day and treatment was maintained for less than 5 days. Schwartz RA et al. [49] confirmed these results and even suggested that higher dosage regimen with 2.7–4 g/kg seem to be more effective in survival outcome. A recent review [111] on 33 pediatric cases of TEN and 6 cases of SJS/TEN overlap showed that therapy with IVIG with a dosage of 0.25–1.5 g/kg for 5 days resulted in 0 % mortality rate and faster epithelization. In conclusion, therapy wth IVIG should be started within the first 5 days and an high-dosage regimen should be preferred (2.5–4 g/kg for adults and 0.25–1.5 g/kg in children divided in 3–5 days).

*Cyclosporine A (Cys A)*: Cys A works through the inhibition of calcineurin, that is fundamental for cytotoxic T lymphocytes activation. In an open trial on cyclosporine in 29 patients with TEN, the use of Cys A for at least 10 days led to a rapid improvement without infective complications [112]. Kirchhof MG et al. [113] retrospectively compared mortality in 64 patients with ED treated either with iv or oral Cys A (3–5 mg/kg) or IVIG (2–5 g/Kg). The authors concluded for a potential beneficial effect of Cys A and a possible improvement in survival compared to IVIG. In conclusion we suggest that therapy with cyclosporine is valuable option with a dosage of 3–5 mg/kg oral or iv for 7 days.

*Anti*-*TNF*-*alpha drugs*:

- Infliximab: chimeric IgG monoclonal anti-TNF-α antibody. It was used with success in different case reports [114–116]. The administration of a single dose of 5 mg/kg was able to stop disease progression in 24 h and to induce a complete remission in 6–14 days. Infliximab was used in cases refractory to high-dosage steroid therapy and/or IVIG.
- Etanercept: monoclonal antibody against the TNF-α receptor. Paradisi et al. [117] described a cohort of ten patients affected by TEN treated with a single dose of etanercept 50 mg sc with a rapid and complete resolution and without adverse events.

Even though there is a strong need for randomized trials, anti-TNF-α drugs, in particular a single dose of infliximab 5 mg/kg ev or 50 mg etanercept sc should be considered in the treatment of SJS and TEN, especially the most severe cases when IVIG and intravenous corticosteroids don’t achieve a rapid improvement.

## Conclusions

EDs are serious and potentially fatal conditions. Their occurrence can be prevented by avoiding drug over-prescription and drug associations that interfere with the metabolism of the most frequent triggers [118]. This is particularly true for patients with many comorbidities and poli-drug therapy, where it is advisable to monitor liver and kidney toxicity and to avoid Vitamin A excess [99]. Genotyping is recommended in specific high-risk ethnic groups (e.g. asiatic) before starting therapies with possible triggers (e.g. HLA-B1502, HLA-B5701, HLA-B5801 and carbamazepine, abacavir, and allopurinol, respectively).

**Fig. 3:**
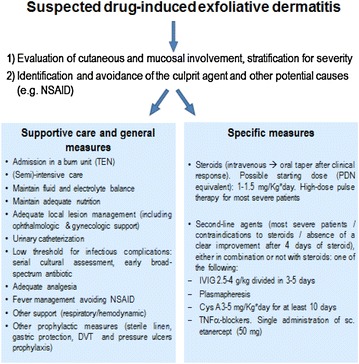
Management of patients with a suspected drug induced exfoliative dermatitis

Once ED has occurred, it has to be managed in the adequate setting with a multidisciplinary approach, and every effort has to be made to identify and avoid the trigger and to prevent infectious and non-infectious complications. Supportive and specific care includes both local and systemic measures, as represented in Fig. 3. For SJS/TEN, corticosteroids are the cornerstone of treatment albeit efficacy remains unclear. Despite improved knowledge of the immunopathogenesis of these conditions, immune-modulatory therapies currently used have not been definitively proved to be efficacious [49, 107], and new strategies are urgently needed.

## Additional files

10.1186/s12948-016-0045-0 Picture of a patient with TEN.
10.1186/s12948-016-0045-0 Picture of a patient with TEN.

## Abbreviations

AGEP: acute generalized exanthematous pustulosis
ALDEN: algorithm of drug causality for epidermal necrolysis
BSA: body surface area
CMV: cytomegalovirus
Cys A: cyclosporine A
DHR: drug hypersensitivity reactions
EBV: Epstein–Barr virus
ED: exfoliative dermatitis
EM(M): erythema multiforme (major)
ENT: ear-nose-throat
G-CSF: granulocyte-colony stimulating factor
GVHD: graft versus host disease
HHV: human herpes virus
HIV: human immunodeficiency virus
HMGB1: high mobility group box 1
HSV: herpes simplex virus
IVIG: intravenous immunoglobulins
LPA: lymphocyte proliferation assay
LTT: lymphocyte transformation test
NSAIDs: non steroidal anti-inflammatory drugs
PCR: polimerase chain reaction
PPI: proton pump inhibitors
PT: patch test
RegiSCAR: European registry of severe cutaneous adverse reactions to drugs
SSSS: staphylococcal scalded skin syndrome
SJS: Steven–Johnson syndrome
TEN: toxic epidermal necrolysis
TNF-α: tumor necrosis factor α
