# Supplementary figures and images for: Drug induced exfoliative dermatitis: state of the art
Source: Clin Mol Allergy. 2016 Aug 22;14(1):9. doi: 10.1186/s12948-016-0045-0 (PMC4993006; doi:10.1186/s12948-016-0045-0)

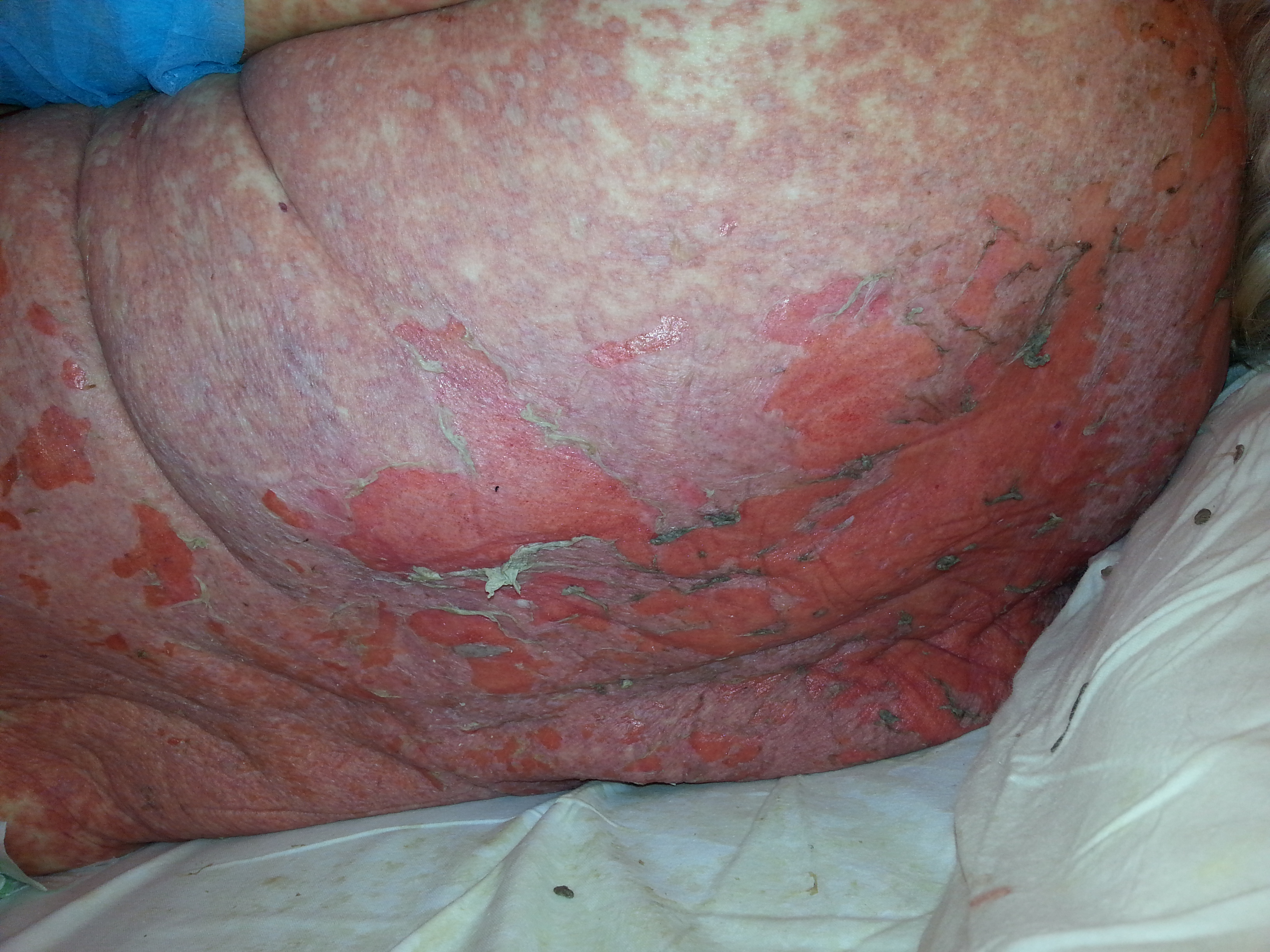

Supplement: Supplementary file 1 — 10.1186/s12948-016-0045-0 Picture of a patient with TEN. [file 12948_2016_45_MOESM1_ESM.jpg]

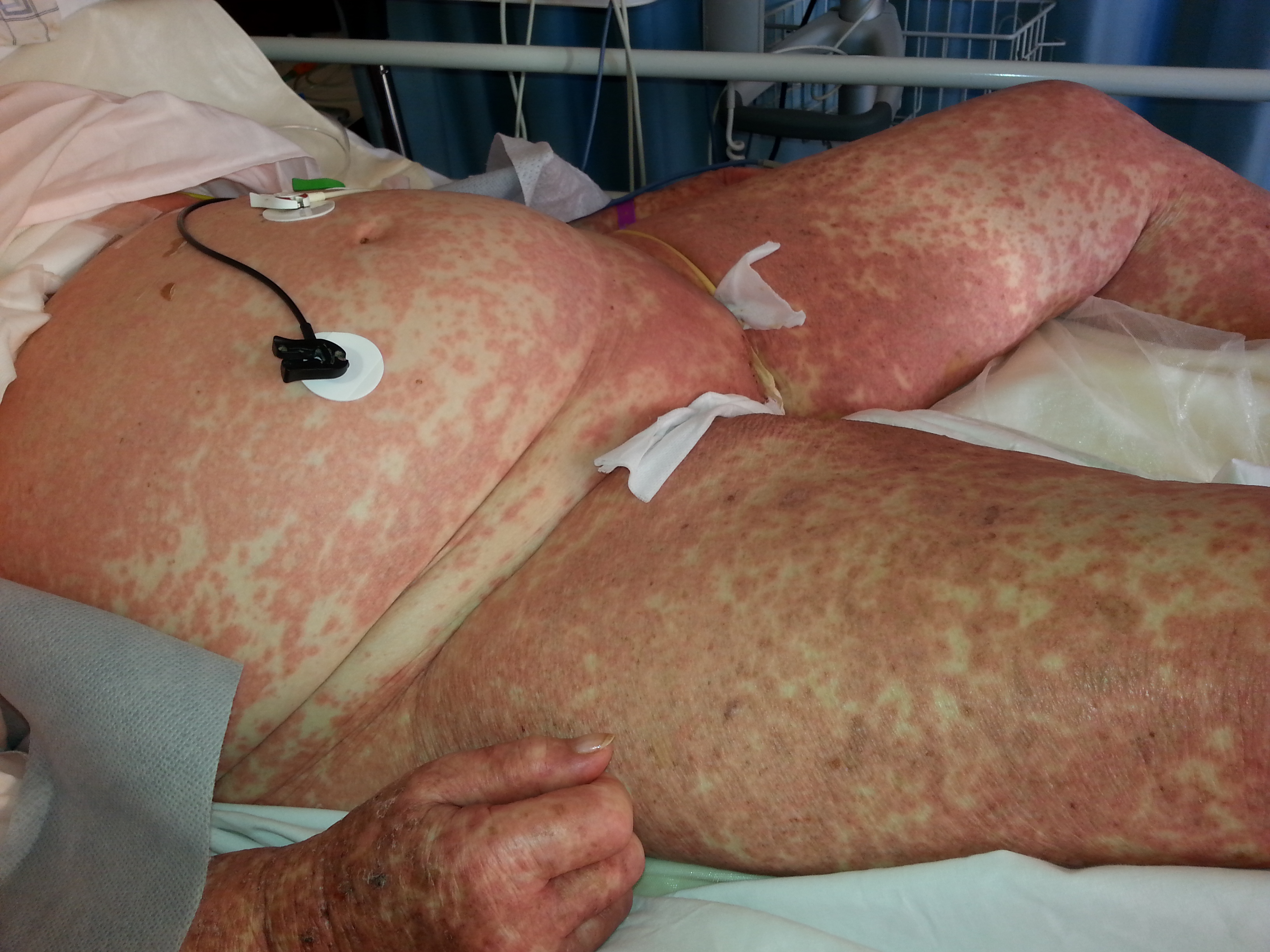

Supplement: Supplementary file 2 — 10.1186/s12948-016-0045-0 Picture of a patient with TEN. [file 12948_2016_45_MOESM2_ESM.jpg]
